# Non-specific impairment of Lung Function on Spirometery in Patients with Chronic Hepatitis-C

**DOI:** 10.12669/pjms.35.2.73

**Published:** 2019

**Authors:** Faisal Faiyaz Zuberi, Bader Faiyaz Zuberi, Tazeen Rasheed, Zunaira Nawaz

**Affiliations:** 1 *Faisal Faiyaz Zuberi, FCPS (Med), FCPS (Pulmo). Ojha Institute of Chest Diseases Dow University of Health Sciences, Karachi, Pakistan*; 2 *Bader Faiyaz Zuberi, FCPS. Dow Medical College Dow University of Health Sciences, Karachi, Pakistan*; 3 *Tazeen Rasheed, FCPS. Dow Medical College Dow University of Health Sciences, Karachi, Pakistan*; 4 *Zunaira Nawaz, FCPS. Dow Medical College Dow University of Health Sciences, Karachi, Pakistan*

**Keywords:** Spirometery, Interstitial pulmonary disease, Hepatitis C, Non-specific impairment of lung function

## Abstract

**Objectives:**

To document frequency of non-specific impairment of lung functions (NILF) in patients of HCV and to compare according to gender, genotype, liver fibrosis score and smoking status.

**Methods:**

Patients of chronic hepatitis C were included after informed consent. Demographic data was recorded, and they underwent baseline investigations, fibroscan, abdominal ultrasound and PFT. Patients were segregated on basis of gender, fibroscan stages and smoking status. NILF was labelled if any two of three criteria are fulfilled (a) FVC < 80% of Predicted, (b) FEV1 < 80% Predicted, (c) FEV1/FVC ≥ 70.

**Results:**

Two hundred thirty four patients were of chronic HCV who fulfilled the selection criteria were inducted in study. These included 49.6% males and 50.4% females. There were 15.0% smokers, 16.2% were ex-smokers while 68.8% were never smokers. NILF was present in 130 (55.6%) out of which 61.5% were female and 38.5% were male (p <0.001), its presence in smokers was 56.2% and in never smokers was 55.3% (p=0.507). Presence of NILF increased with Fibroscan stages from F1 to F4 (p <0.001).

**Conclusions:**

NILF pattern on spirometry with normal chest radiograph is common among HCV patients. It was found more common in females and frequency increased progressively with fibro scan stages.

## INTRODUCTION

Infective viral hepatitis is a worldwide health problem. Hepatitis B and C have greater impact on health and sufferings due to their chronicity and life-threatening complications. The Global Burdon of Disease quantifies health impairment by Disability Adjusted Life Years (DALY) which takes into account the non-fatal outcomes and pre-mature death.^1^ The greatest number of deaths and DALY attributes for viral hepatitis were reported from East and South Asia.^2^ Hepatitis C (HCV) prevalence in Pakistan has been reported at 4.9-13.0% depending on the method and area screened.^3^,^4^ Although main disease burden and complications are liver related in this infection, but it has been reported to have several extra-hepatic manifestations too. These include thyroiditis, linchin planus, glomerulonephritis, cryoglobinemia and interstitial pulmonary involvement. 5,6

Chronic HCV causes inflammation leading to its hepatic and extra-hepatic manifestations by poorly understood mechanisms. Hepatocytes, Kupher and stellate cells are known to produce substances that produce inflammatory responses directly and via macrophages that are responsible for such inflammatory response.^7^ Inflammation induced by HCV plays significant role in hepatic and extra-hepatic manifestations of chronic HCV infection.^7^ Information is lacking on complex mechanisms causing HCV associated extra-hepatic manifestations as this could shed light on disease progression, prognosis and help in development of new strategies for treatment. Moreover it is important to realize that successful treatment of HCV does not always result in resolution of extra-hepatic manifestations and complications.^8^

Recently HCV is also implicated in development of pulmonary dysfunctions including FEV1, FVC and FEV1/FVC ratios in large Third National Health and Nutrition Examination Survey (NHANES III).^9^ There are several reports that HCV infection leads to accelerated decline in pulmonary functions test (PFT) parameters and others have shown high prevalence of HCV in COPD patients.^10^ Derangements in PFT is recognized as independent risk for mortality.^11^ This study was designed to see changes in PFT on spirometery in patients suffering from chronic HCV infection. Simple spirometry results are interpreted as one of the four interpretation patterns:

- Normal
- Non-specific Impairment in Lung Function (NILF)
- Obstructive and
- Obstruction with reduced FVC.

NILF pattern and Obstruction with reduced FVC both patterns may suggest ‘restriction’ but need static lung volume measurements to prove whether restriction is present or not. Literature search shows scanty data on Non-specific Impairment of Lung Functions (NILF) in patients of HCV and there is no published report on this topic from Pakistan. The study was conducted with objective to determine frequency of NILF in HCV patients and to compare it on basis of smoking status and liver fibrosis scores. It will thus help in better identification and treatment of such patients.

## METHODS

### Sample Size

Sample size was calculated for proportions using PASS version 15.0 software, and using reported frequency of FEV1 abnormalities of 25% in patients of HCV.^12^ It was also calculated for one Proportion using parameters of P0 = 0.5; P1 = 0.25; Power = 0.95; Alpha = 0.01; n = 65. Taking into account of 20% dropout, the Dropout Inflated Sample Size the number was 82.

### Inclusion Criteria

Patients of Chronic HCV attending liver clinic were included for study after taking informed consent.

- Chronic HCV was defined as having HCV infection for more than 6 months
- Vibration Controlled Transient Elastography (VCTE) median between 5.0-40.0 kPa on Fibroscan with IQR/M of ≤20%
- Normal Chest X-ray PA View

### Exclusion Criteria

Known patients of respiratory diseases including asthma, COPD, interstitial pulmonary fibrosis

- Pulmonary tuberculosis
- Cardiac failure
- Pregnancy
- Decompensated Cirrhosis
- Ascites

### Operative Definition

1. ***Non-specific Impairment of Lung Function:*** (NILF) was labelled if any two of the following criteria are fulfilled.
  - FVC < 80% of Predicted
  - FEV1 < 80% Predicted
  - FEV1/FVC ≥ 70
2. ***Chronic Hepatitis C:***
  - Presence of anti-HCV for ≥ six months
3. ***Vibration-Controlled Transient Elastography (VCTE) Stages:***
  - F1 2.0-7.9 kPA
  - F2 8.0-9.9 kPa
  - F3 10.0-13.9 kPa
  - F4 ≥ 14.0 kPa

All selected patients underwent standard workup for HCV including CBC, CXR-PAV, LFT, Serum Albumin, INR, Ultrasound Abdomen, Quantitative PCR and Genotype. VCTE was done using M-Probe in patients of BMI < 30 and using XL-Probe in patients with BMI of ≥ 30. Ten readings were taken, and median value was used for analysis. Child Turcot Pugh’s (CTP) and MELD Scores were calculated. All patients underwent standard Spirometry Test using Spirobank-II. Three attempts were made and best of three was used for analysis.

All parameters were compared according to Age Groups, Genotype and Fibroscan Stages using Chi-Square test. Smoking and pack years were stratified and analysed for their effect using Student’s‘t-test’. P value of ≤0.05 was set as significant.

## RESULTS

Two hundred thirty four patients were of chronic HCV who fulfilled the selection criteria were inducted in study that exceeded our sample size of 82. These included 116 (49.6%) males and 118 (50.4%) females. Mean age ±SD of males was 48.37 ±16.38 years and that of females was 41.69 ±14.97 years. Age of males was significantly more than that of females (p=0.001; df=232; 95% CI -10.72 to -2.64). BMI of males was 31.90 ±25.59 kg/m^2^ and of females was 32.0 ±35.58 kg/m^2^. No statistically significant difference was found in BMI among both genders (p=0.982; df=232; 95% CI -7.90 to 8.08). There were 35 (15.0%) smokers and 38 (16.2%) were ex-smokers while 161 (68.8%) were never smokers. Among the smokers the median quantity of smoking was 13.5 pack years (range=59; IQR=23) and median duration in years was 10 years (range=34; IQR=11). Details are given in Table-I. The breakup of HCV Genotypes (GT) included GT1 40 (17.1%), GT2 17 (7.3%), GT3 163 (69.7%) & GT4 14 (6.0%). Patients’ data was recoded into a new variable (NILF = Yes/No) based on diagnostic criteria of NILF as given in methods section. Frequency of patients fulfilling NILF criteria was 130 (55.6%). Frequency of NILF in females (61.5%) was found to be significantly more than that in males (38.5%), (p <0.001) details in Table-II.

**Table-I:** Demographic details of studied patients.

	Female (n=118)		Male (n=116)		Total (n=234)	

	Mean	SD	Mean	SD	Mean	SD
Age in Years	41.69	14.97	48.37	16.38	45.00	16.00
BMIs	24.35	5.49	24.90	5.39	24.62	5.44

	*n*	*%*	*N*	*%*	*n*	*%*

Smoker	13	11.0	22	19.0	35	15.0
Ex-Smoker	3	2.5	35	30.2	38	16.2
Never Smoker	102	86.4	59	50.9	161	68.8
Smoke Years (Median)					10	
Smoke Pack Years (Median)					13.5	

**Table-II:** Comparison of different variables with NILF.

		NILF					P value

		Yes		No		Total	

		Count	Row N %	Count	Row N %		
Gender	Female	80	67.8%	38	32.2%	118	<0.001
	Male	50	43.1%	66	56.9%	116	
Smoking Status	Never Smoker	89	55.3%	72	44.7%	161	0.507
	Ex/Current Smoker	41	56.2%	32	43.8%	73	
Genotype	GT1	17	42.5%	23	57.5%	40	0.134
	GT2	7	41.2%	10	58.8%	17	
	GT3	98	60.1%	65	39.9%	163	
	GT4	8	57.1%	6	42.9%	14	
VCTE Stage	F1	5	6.8%	69	93.2%	74	<0.001
	F2	13	35.1%	24	64.9%	37	
	F3	26	81.3%	6	18.8%	32	
	F4	86	94.5%	5	5.5%	91	

Mean values ± SD of FVC Pred, FEV1 Pred & FEV1/FVC in our study were 2.99 ±0.61, 2.52 ±0.52 & 64.24 ±17.52 respectively. Comparing means of all above PFT parameters by patients who smoked, ex-smokers & never smoked by one-way ANNOVA significant statistical difference were found in FVC Pred & FEV1 Pred but not in FEV1/FVC. Table-III.

**Table-III:** Comparison of FVC Pred, FEV1 Pred & FEV1/FVC with smoking status of Never Smoker, Ex-Smoker & Current Smoker using ANNOVA.

		Sum of Sq	df	Mean Sq	Mean	SD	F	Sig.
FVC_PRED	B/W Groups	5.68	2	2.844	2.99	0.61	8.099	.000
	W/I Groups	76.20	217	.351				
	Total	81.89	219					
FEV1_PRED	B/W Groups	3.36	2	1.684	2.52	0.52	6.667	.002
	W/I Groups	54.82	217	.253				
	Total	58.19	219					
FEV1/FVC	B/W Groups	444.43	2	222.219	64.24	17.52	.722	.487
	W/I Groups	71086.74	231	307.735				
	Total	71531.17	233					

Patients who were current and ex-smokers were combined to create a new variable with only two values, that who never smoked and those who were ex/current smokers. It was found that 161 (68.8%) patients never smoked while 73 (31.2%) patients were ex/current smokers. Assessing frequency of NILF with smoking status using cross-tabulation it was observed that frequency of NILF in patients who never smoked was 89 (55.3%) while those who smoked was 41 (56.2%). The difference in frequency of NILF among never smokers and ex/current smokers was not significant (p=0.507). Table-II.

NILF presence was analysed with HCV GT and showed highest percentage of NILF in GT3 (60.1%) followed by GT4 (57.1%), GT1 (42.5%) & GT2 (41.2%). No statistically significant difference was found in frequency of NILF according to GT (p=0.134). VCTE staging was analysed with presence of NILF and it showed that VCTE F4 stage was associated with highest presence of NILF of 94.5% and lowest in F1 which increased sequentially from F1 to F4. The difference was statistically significant (p<0.001). Table-II.

## DISCUSSION

Our study showed two important findings first that NILF is more common in females and second its frequency is similar in those who never smoked and ex/current smokers. These are very important findings with significant impact on clinical practice. Findings that NILF is as common in HCV as in smokers is very significant. Other pulmonary disorders have also shown to be associated with HCV. Several studies have demonstrated increased incidence of idiopathic pulmonary fibrosis (IPF) in patients with HCV.^7^,^12^,^13^ Arase Y et al demonstrated this effect in large cohort of patients with HCV and HBV.^13^ They showed that IPF was more common in HCV as compared to in HBV infection. There are also some studies that do not show restrictive pattern association between HCV and pulmonary disease but rather association of asthma with HCV.^9^

HCV is a hepatotropic, non-cytopathic virus that can cause hepatitis, cirrhosis, and hepatocellular carcinoma (HCC).^14^,^15^ It is also known to cause many extra-hepatic manifestations including auto-immune thyroid disease.^16^ Impaired lung functions have also been demonstrated in HIV infection and fatty liver disease.^17^,^18^ HCV produces many inflammatory mediators including TNF-α, TGF-β, interleukin (IL-6), and IL-8 by altering cellular signalling and genetic imbalances.^19^ It has been shown that IL-8 also plays important role in pathogenesis of lung inflammation resulting in impairment of lung functions and fibrosis.^20^ In a study by Chen WC et al they showed that HCV induced IL-8 activation leads to COX-2 activation through MAPK pathway.^14^ This in turn plays important role in HCV proliferation, pulmonary impairment and HCC development.^14^

Association of idiopathic pulmonary fibro (IPF) is well documented in HCV and many researchers are of opinion that it is under reported and under diagnosed.^12^,^21^ Up to 75% risk of sub-clinical involvement of lungs has been reported in HCV.^12^ More-over significant increase in cellular content with preponderance of polymorphonuclear cells in BAL in patients with HCV has also been reported, authors were of view that these inflammatory cells cause occult pulmonary inflammation that could lead to IPF.^22^ Apart from its aetiological role in IPF, HCV has also been suggested to compromise pulmonary function in patients with chronic obstructive pulmonary disease (COPD) or asthma.^10^,^23^,^24^ Asthmatic patients with HCV infection have shown better responses to corticosteroid therapy under concurrent anti-viral therapy, moreover, worsening of pulmonary function was halted in HCV-infected patients with COPD, who responded to anti-viral therapy.^23^-^25^

### Limitations of the study

We did not do Diffusion lung capacity for carbon monoxide (DCLO), corrected DCLO (DLCO/VA) and static lung volumes and High-resolution computer tomography (HRCT) of patients in our study to prove and categorize restrictive lung disease. The first three ones require expensive instruments that are scarcely available and as we were trying to document the NILF, these investigations were not required.

## CONCLUSIONS

NILF pattern on spirometry with normal chest radiograph is common among HCV patients. It was found more common in females and frequency increased progressively with fibro scan stages.

### Authors Contribution

**FFZ** did PFTs, wrote manuscript, gave final approval of manuscript.

**BFZ** designed and conceived the study & did statistical analysis.

**ZN & TR** collected data, literature search and did initial draft writeup.
